# The world's richest tadpole communities show functional redundancy and low functional diversity: ecological data on Madagascar's stream-dwelling amphibian larvae

**DOI:** 10.1186/1472-6785-10-12

**Published:** 2010-05-12

**Authors:** Axel Strauß, Erik Reeve, Roger-Daniel Randrianiaina, Miguel Vences, Julian Glos

**Affiliations:** 1 Zoological Institute, Technical University of Braunschweig, Spielmannstr. 8, 38106 Braunschweig, Germany; 2 Département de Biologie Animale, Université d'Antananarivo, Antananarivo 101, Madagascar; 3 Zoological Institute, University of Hamburg, Martin-Luther-King Platz 3, 20146 Hamburg, Germany

## Abstract

**Background:**

Functional diversity illustrates the range of ecological functions in a community. It allows revealing the appearance of functional redundancy in communities and processes of community assembly. Functional redundancy illustrates the overlap in ecological functions of community members which may be an indicator of community resilience. We evaluated patterns of species richness, functional diversity and functional redundancy on tadpole communities in rainforest streams in Madagascar. This habitat harbours the world's most species-rich stream tadpole communities which are due to their occurrence in primary habitat of particular interest for functional diversity studies.

**Results:**

Species richness of tadpole communities is largely determined by characteristics of the larval habitat (stream structure), not by adult habitat (forest structure). Species richness is positively correlated with a size-velocity gradient of the streams, i.e. communities follow a classical species-area relationship. While widely observed for other taxa, this is an unusual pattern for anuran larvae which usually is expected to be hump-shaped. Along the species richness gradient, we quantified functional diversity of all communities considering the similarity and dissimilarity of species in 18 traits related to habitat use and foraging. Especially species-rich communities were characterised by an overlap of species function, i.e. by functional redundancy. By comparing the functional diversity of the observed communities with functional diversity of random assemblages, we found no differences at low species richness level, whereas observed species-rich communities have lower functional diversity than respective random assemblages.

**Conclusions:**

We found functional redundancy being a feature of communities also in primary habitat, what has not been shown before using such a continuous measure. The observed species richness dependent pattern of low functional diversity indicates that communities with low species richness accumulate functional traits randomly, whereas species in species-rich communities are more similar to each other than predicted by random assemblages and therefore exhibit an accumulation of stream-specific functional traits. Beyond a certain species richness level, therefore, stream-specific environmental filters exert influence whereas interspecific competition between species does not influence trait assemblage at any species richness level.

## Background

Diversity is an important characteristic of communities, with paramount influences on ecosystem properties [1]. A wide range of measures have been applied for quantifying diversity, the simplest of which is species richness (SR): the number of species in a community [2]. SR assumes species as comparable, distinct entities of similar ecological importance. However, differences between species regarding ecological traits may range from almost ecologically similar to very different. Therefore, in recent years the focus has turned from SR towards functional diversity, which considers components that influence ecosystem function rather than taxonomic units [3]. The general concept of species function being more important than species richness has been shown in several studies, e.g. in predicting plant community resistance [4] and plant biomass accumulation [5].

A common approach in measuring functional diversity is classification of functional species groups [3,6,7]. This requires an *a priori *classification of species resulting in a discontinuous and, therefore, less accurate measure of functional diversity [8] than a continuous measure (FD) defined by Petchey & Gaston [9,10]. Additionally, it can be difficult to fit species varying ecomorphologically in a complex multidimensional space into predefined groups defined by a limited number of characters [e.g. [11]]. Alternatively, FD compiles a variable range of ecological characteristics of species and is regarded as a very powerful measure of functional diversity [12].

Patterns between changes in functional diversity and SR provide information on the relative contribution of a species' ecological function to the sum of ecological functions of the community. Therefore, if functional diversity and SR show a one by one relationship, all species are different and contribute equally. Deviations from this pattern occur with differences in species contribution, e.g., if SR changes but functional diversity remains constant, the additional or diminished species do not exhibit unique ecological traits and can be considered as functional redundant [13]. Patterns of functional redundancy were identified using FD in mammal, bird [14], and amphibian communities [15,16]. However, these findings of functional redundancy are so far only related to anthropogenically disturbed landscapes. Comparing FD of observed and random assemblages can be used to test for non-randomness, which can highlight general processes of community assembly [17], such as competition or environmental filtering [18]. Communities harbouring a large number of species are likely to contain species that are redundant in their ecological traits. The question of functional diversity and redundancy in species communities is therefore of particular interest when facing taxonomic groups that are rich in species. Tropical anuran communities represent an appropriate model as they are known to be remarkably rich but still taxonomically ascertainable. Studies on tropical frogs often focus on the ecology of adults [19-23], and have shown that SR can be predicted by environmental variables [21,24], and that species specific habitat requirements may be overlaid by stochastic processes [23,25]. Minor attention has focused on functional diversity and functional redundancy in tropical amphibians, although Ernst et al. [15,16] showed that functional redundancy can be found in disturbed tropical frog communities and the classical measure of species richness fails to reflect the real dimensions of biodiversity.

Of the available amphibian community studies, only a few included larval stages [26-28]. Even less attention was given to diversity patterns of the tadpole communities themselves [29,30], although in pioneering studies different habitat variables were found to be possibly related to SR of tadpole communities [22,27]. There are no published data on functional diversity in tadpole communities and the validity of SR as an adequate measure of diversity remains to be verified.

This is especially true as there are several ways tadpole communities might influence ecosystem function. There is evidence that e.g. by moving sediment and feeding on primary algae producers, tadpoles can alter algae abundance, composition, and chlorophyll *a *level and therefore net primary production in stream ecosystems [31]. Furthermore, due to their influence on basal resources e.g. removing sediments and exposing periphyton, they affect other primary consumers [32]. Tadpoles can therefore affect stream ecosystem structure and function [31,32] depending on where they live in the stream and how they forage. This might be especially true if some higher trophic levels are missing in the ecosystem.

The remarkable backlog of tadpole community studies may have been caused by identification difficulties, especially in species-rich tropical communities where the ecological importance of tadpole communities may be paramount [32-35]. Madagascar, regarded to be one of the most important hotspots for biodiversity conservation [36] harbours over 400 fully endemic frog species [37,38]. Even if many of these species are yet undescribed scientifically, a near-complete database of genetic markers exists [38]. This allows application of molecular identification methods to identify tadpoles to species [39], and allows community studies of tadpoles in an area known to harbour rich frog communities [37,40].

Here, we report on the SR and functional diversity of stream communities of tadpoles in Ranomafana National Park (RNP) in eastern Madagascar as determined by DNA barcoding, and on the environmental variables that might influence these measures of diversity. We addressed three main questions: (1) are stream tadpole communities in Madagascar as rich as expected given the highly diverse amphibian communities, (2) is SR predictable by either adult or tadpole related environmental variables, and (3) does the functional diversity measure expose patterns of diversity that are not detectable by SR and point to general rules of species' trait assemblage?

## Results

### Habitat ordination

**Figure 1:**
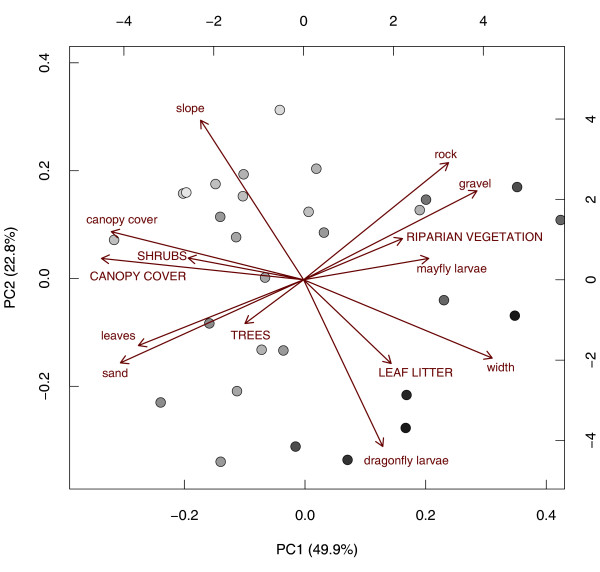
**Principal component biplot and SR pattern**. Principal component biplot showing PC1 and PC2. Vectors show the environmental variables, tadpole habitat characteristics are in lower case, adult frog habitat parameters are in capital letters. Filled circles represent study sites (streams) that are plotted according to their scores (pay attention to scales). Symbols are coded by continuous grey shading showing low SR (light grey) to high SR (dark grey).

We could summarise the 14 original habitat variables to three PC factors (according to the scree plot) with PC1 representing 49.9%, PC2 22.8%, and PC3 13.0% of the original variation. Bootstrapping-eigenvector method highlighted these habitat variables as significantly contributing to PC1: stream *slope *(-), stream *canopy cover *and forest *CANOPY COVER *(-), *SHRUBS *(-), and the stream microhabitats *sand *(-) and *leaves *(-) as well as *rock *(+) and *gravel *(+), stream *width *(+), *mayfly larvae *(+) and *dragonfly larvae *(+) (Figure 1). For PC2 this were stream *width *(-), stream microhabitat *sand *(-), and *dragonfly larvae *(-) as well as stream *slope *(+), and the stream microhabitats *rock *and *gravel *(+) (Figure 1). The strongest contributions to the PC3 come from forest *LEAF LITTER *(-), forest *CANOPY COVER *(-), and forest *TREES *(+), however, the results were not significant.

PC1 therefore represents a gradient from smaller streams with a generally dense canopy cover and a high proportion of microhabitats consisting of leaf and sand substrate towards larger streams with a higher proportion of gravel and rock substrate and higher invertebrate larvae abundances. This gradient is highly significant and positively correlated with SR (multiple linear regression, F_2,26 _= 50.75; R^2 ^= 0.80, p_model_<0.001; p_PC1_<0.001). PC2 represents a gradient from larger, slow-moving and sandy streams with high abundance of dragonfly larvae towards small and rocky streams with steep slopes. This gradient is highly significant and negatively correlated with SR (p_PC2 _<0.001). As the third PC was not correlated to SR, it was removed from the model in order to find the minimal adequate model.

We overlaid a PCA biplot with SR (Figure 1), which illustrates a gradient of increasing SR from the top left corner to the bottom right corner (for alternative graphical illustration see additional file 1: regressions of PCs vs SR). Accordingly, SR increases mainly with stream *width *and *dragonfly larvae*, and decreases with stream *slope*, stream *canopy cover *and forest *CANOPY COVER*. The proportion of specific stream microhabitats (i.e. stream substrates) did not have a major impact on SR.

### Functional diversity (FD)

**Figure 2:**
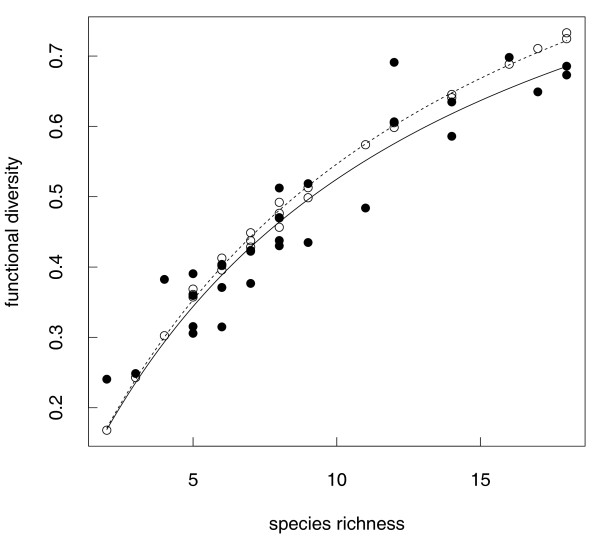
**Non-linear regression of SR and observed and predicted FD**. Comparison of predicted (black circles, dashed line) and observed functional diversity (FD, filled dots, continuous line) along species richness. Predicted FD calculated from simulated random assemblages. Lines show the fitted non-linear regression.

Applying a polynomial regression model, we found a highly significant positive correlation between SR and FD (F_1,27 _= 209, R^2 ^= 0.89, p_model_<0.001, p_SR_<0.001). Although the increase of R^2 ^(0.89 vs. 0.91) was low, the included quadratic term still significantly contributed to the model (F_2,26 _= 124.3, R^2 ^= 0.91, p_model_<0.001, p_SR_<0.001, p_SR^2 _= 0.028, Figure 2). This correlation is therefore not linear but shows a decreasing slope with higher SR. This pattern expresses an increase in functional redundancy of tadpole species with increasing SR of the stream community.

The predicted FD of random communities showed a similar pattern (Figure 2). Due to violation of independence of the residuals we could not fit a linear model for predicted FD and therefore applied Michaelis-Menten with parameter estimates for maximum FD and the SR of communities with mean FD to compare curve progression of both predicted and observed data. This non-linear regression model and t-tests applied on the parameter estimates show that the maximum FD for the observed communities (FD = 1.11 ± 0.07) was significantly lower than the maximum predicted FD for the random communities (FD = 1.21 ± 0.08; t-test, t = -9.501, df = 54, p < 0.001). There was no difference in SR values at a mean FD between observed (S = 11.12 ± 1.32) and predicted communities (S = 12.06 ± 1.40; t-test, t = 6.275, df = 54, p = 1). These tests and the graphical evaluation (Figure 2) describe a pattern of low functional diversity but only in species-rich communities.

## Discussion

### Madagascan streams as tadpole hotspots

Madagascan rainforest streams harbour the world's most species-rich tadpole communities. In 30 m sections of RNP mid-elevational rainforest streams in the current study, species richness (SR) of tadpole assemblages varied from two to 18 species, with an average of nine species and a total number of 36 species. At another site in Madagascar, An'Ala rainforest in central eastern Madagascar, an even higher number of species was reported with a maximum of 28 species in a 50 m stream section [40]. Distinctly lower numbers of species were reported from tropical streams in Brazilian forests (1-8 species, [41]; 1-9 species, [22]; 2-10 species, [27]). Equally remarkable is that the Malagasy stream tadpole communities can be impressively rich in specimens. We detected up to 1,100 tadpole individuals per 30 m of stream which emphasises their high importance for the Malagasy stream ecosystems and claims for explanations beyond a simple correlation with frog species richness. In fact, the number of frogs in both RNP and the Mantadia-Analamazaotra area (including An'Ala forest) is around 100 species which is very high but not markedly different from other tropical amphibian hotspots [38]. However, Madagascan rainforests show a higher proportion of stream-breeding frog species than other tropical amphibian communities which often contain many pond breeders. In general, Madagascan rainforests along the geographically steep eastern escarpment do not offer many pond breeding habitats which explains the low number of pond breeding species. There is little information available on species breeding in phytotelmata, tree holes or foam nests on the forest floor but we assume their relative frequency is low.

We also consider the virtual absence of fish possibly a main reason for the exceptional tadpole diversity and abundance in Madagascan rainforest streams. Both RNP and An'Ala, and Madagascan rainforest streams in general, are exceptional among tropical rainforest streams in their remarkably low density and diversity of fish. In most RNP streams, only the eel (*Anguilla *sp.) occurs in detectable although very low numbers.

### Tadpole diversity is dependent on stream size and velocity

Tadpole diversity is not explained by those habitat variables that are important for adult frogs. Neither the forest structure around a stream nor its streamside vegetation structure correlated significantly with tadpole SR. This shows that tadpole diversity is not limited by environmental filters that affect adults and that might cause streams not being used for breeding although they might represent suitable habitat for tadpoles.

In contrast, those habitat variables that directly act on tadpoles explain tadpole diversity very well. Based on the results of the PC and regression analyses, diversity concerning both, SR and FD, increases along a stream size-velocity gradient, i.e. it was highest in slow moving (low *slope*), large streams, with open canopy cover, and a high abundance of dragonfly larvae. The proportion of specific ground substrates within the streams was not important.

The observed stream size - SR dependency follows a general ecological pattern, i.e. the species-area relationship that is found very commonly for a wide variety of taxa and types of ecosystems, and only few exceptions exist [e.g. [42]]. In fact, the species-area relationship is often referred to as the closest thing to a rule in ecology [43]. It states that along a gradient of ecosystems of increasing size, the numbers of species inhabiting those ecosystems increase, in general rapidly at first, and then more slowly for the larger ecosystems. However, many tadpole communities and their ecosystems, e.g. streams and ponds, are among the exceptions. For stream habitats studies on the dependencies of stream size and tadpole diversity are inconclusive. Both positive and negative continuous relationships are found, i.e. the most diverse communities are in the largest [19] or in the smallest streams [22,27]. For tadpole communities of tropical ponds, as a general pattern, tadpole SR increases with increasing pond size, but beyond a certain size, ponds are permanent (vs. temporary), and an increasing number of fish eliminate tadpoles. This results in medium-sized water bodies harbouring the highest number of tadpole species [44]. Our rainforest stream data are generally consistent with the predictions derived from these studies on ponds, although there is no peak in SR at an intermediate stream size. All the streams in the current study are permanent, and as a peculiarity, fish are not an important factor for tadpole survivorship. Therefore, factors that limit SR beyond a certain water body size may thus not be effective in RNP tadpole communities.

The increase of SR along the stream size-velocity gradient cannot be attributed unambiguously to a higher number of different microhabitats in larger habitats. All microhabitats, i.e. ground substrates, were present in all streams, and their respective proportions were not significantly correlated to SR. High stream velocity, in contrast, might be a factor limiting SR. Of the species occurring in the RNP region, only a few have morphological adaptations to strong currents [11,45] and high currents might prevent some species from colonising streams. In general, selective pressures caused by stream current can be hypothesised to be stronger in fast-running portions of the streams, where tadpoles not adapted to such conditions will be washed away during high flow levels after heavy rain. In contrast, in slow-moving stretches, tadpoles adapted to stronger currents will be able to survive, even if they may suffer from increased competition with other larvae better adapted to these conditions.

### FD and functional redundancy are dependent on species richness

We found both functional redundancy and low FD in tadpole communities of Madagascan rainforest streams. The presence of functional redundancy indicates an overlap in the traits of species within a community. While this can be interpreted as dispensability of some species, it is also a buffer to ensure community resilience [46,47]. Patterns of redundancy have been reported when using functional groups e.g. for bat [48], plant [49-51], bacterial soil communities [52], and coastal marine assemblages [53]. However, the functional group approach highlights only some functions of species and disregards a possible wide range of others. As focusing on few traits likely leads to findings of patterns of functional redundancy [9,49], we used a continuous measure using 18 morphological traits relating differently to habitat use and feeding ecology (Table 1) to include a wide range of species functions that can influence stream ecosystem structure and function.

**Table 1:** Ecological traits and the representing morphological traits of tadpole species used for calculating functional diversity

Ecological trait	Morphological trait	Type of data
feeding and ability of habitat use by shape of oral disc	mouth opened	binary
	umbelliform	binary
	suctorial	binary
	generalised or small but with keratodonts	binary
	reduced	binary
	generalised	binary
		
feeding type (e.g. filterer, grazer, carnivore) represented by jaw sheaths shape	generalised	binary
	keratinised, vertical bars	binary
	poorly keratinised	binary
	transformed in a three sporn-shaped papillae	binary
	transformed in bow-net structure	binary
	absent	binary
		
feeding type	number of keratodonts rows	continuous
		
habitat use (adaptations to water current)	number of papillae	continuous
	relative oral disc width	continuous
	relative tail muscle height	continuous
	relative tail length	continuous
habitat use (use of the water column)	eye position	binary

Previous identification of functional redundancy quantified by continuous FD were generally attributed to agricultural landscapes or anthropogenic disturbed sites [14,15]. We here show, however, that functional redundancy is also an attribute of communities in primary freshwater stream habitats. Whereas a linear but only slightly increasing relationship between SR and FD shows a continuous pattern of redundancy, the curvilinear shape observed for tadpole communities shows that functional redundancy depends on the level of SR, with the highest functional redundancy assigned to species-rich communities in large streams. We here face the world's most species-rich communities, however, compared to many other ecological systems, the absolute number of species is relatively low. For example, functional diversity studies on plant communities may include 11 to 75 species per community [50], a study on deep sea nematode assemblage up to 80 species per site [54] and up to almost 480 species per site in a reef fish study [51]. Identification of potential SR-FD relationships may be difficult using only a low SR or low range of SR [18,51]. The range of SR of tadpole communities is obviously sufficient to detect patterns, as indicated both in the curvilinear FD curve and the species richness dependent functional redundancy (discussed below). The fact that functional redundancy and low FD of tadpole communities are not very pronounced compared to other studies [e.g. [51]] still supports the need of a sufficient range of SR for studies on FD [51]. Facing adult frog plus tadpole traits and using about twice the number of species as in the present study (up to 39 per site and 55 in total) showed quite clear patterns of redundancy in West Africa [15]. However, studying a large number of species often implies a large geographical study area [e.g. [48,51]]. Consequently, using all ecological species traits available in the whole geographical range for random FD calculations and thereon depending comparisons with observed data may lead to patterns of e.g. low FD that are difficult to assign to either ecological or geographical filters, or both.

We could show that with increasing SR, the FD of tadpole communities was increasingly lower than the FD of randomly assembled communities of similar SR levels. This difference between observed and predicted FD values shows low FD and indicates that members of species-rich communities were more similar to each other than expected by random assembling. Low FD was observed in bird [18], plant [50] and reef fish communities [51], however, only the latter showed a similar SR-dependent pattern. This is an indication of SR dependent environmental filtering [18] and whereas up to a certain level of SR the assemblage of different traits of tadpoles is random, in richer communities stream-specific traits accumulate. As discussed above, low FD in tadpole communities is statistically significant but still not as pronounced as e.g. in reef fish communities [51]. However, differences in the geographical ranges in the studies and the resulting difficulties of interpretation complicate the comparison of studies. If competition is a shaping force, species characterised by dissimilar traits would form a community, resulting in high values for FD [18]. This was not the case for any level of SR. As we used traits for calculation of FD related to habitat use and foraging of tadpoles, we conclude that interspecific competition for space and food does not influence the composition of tadpole communities in Madagascan rainforest streams. It is habitat characteristics of the stream and/or the (non-)availability of food that filters specific traits and therefore specific species from communities, at least in species-rich large streams.

## Conclusions

In summary, (1) SR of Madagascan stream tadpole communities generally follows a species-area relationship leading to the worlds highest number of tadpole species, (2) evidence from these communities shows occurrence of functional redundancy in primary freshwater habitats, and (3) environmental filtering but not interspecific competition may be a major factor influencing assembly of tadpole communities. We could prove both findings of functional redundancy and low FD associated with high SR for reproducibility by repeated sampling of streams in RNP in 2008 (own unpublished data). These results highlight the potential of FD to provide insights into the under-investigated communities of larval stages of anurans. We see particular promise in future studies that integrate these findings with further community characteristics such as food web structures, and we predict these will help elucidating the fundamental processes that structure amphibian communities.

## Methods

### Study sites

We conducted fieldwork during the rainy season, February and March, 2007, in one of the centres of amphibian species richness in Madagascar, the Ranomafana National Park (RNP; 21°16'S; 47°25'E; 500 - 1500 m a.s.l.). RNP covers over 40,000 ha of mid-elevational rain forest and harbours over 100 frog species [38]. Due to varied topography and high average annual rain fall of 2,000 mm, RNP has numerous streams which are generally permanent, with broad variations in abiotic and biotic characteristics.

Study sites were represented by 30 m sections of 29 permanent streams without direct upstream link between any two sites and comprised various types of streams in a similar number. Streams of obvious different habitat characteristics were sampled alternately to avoid a time effect on sampling results for specific stream types. Of each 30 m section, three groups of habitat variables were recorded: (1) characteristics of the stream, representing habitat relevant for tadpoles, (2) characteristics of the adjacent forest, representing habitat relevant for frogs, and (3) structure of riparian vegetation, which is relevant for breeding activities.

#### Aquatic habitat

We laid transects consisting of adjoining 1 × 1 m quadrates crossing the stream perpendicularly. Starting at the downstream end of the sampling section, we recorded stream variables of 10 diagonal transects at intervals of 2 m, thus covering 33% of the area of the sampling section. We recorded stream width (m) in the centre of each of these transversal transects, and averaged (variable *width*). Stream velocity was analysed by measuring the stream *slope *(m), i.e. the difference in altitude between the upstream and the downstream end of the 30 m stream section. We recorded in each 1 × 1 m quadrate the *canopy cover *(coded as 0 = absent and 1 = present, and averaged), and the proportion of each microhabitat type (%). We defined microhabitat types based on ground substrate: *leaves *(organic material), *sand *(very finely grained up to ~3 mm grain size), *gravel *(grain size 3 mm to 25 cm), and *rock *(> 25 cm). As biotic habitat variables, we sampled *dragonfly *(Anisoptera) and *mayfly *(Ephemeroptera) *larvae *which we conserved in 99% ethanol immediately in the field to avoid possible predation events within the sampling containers.

#### Terrestrial habitat

We recorded forest habitat characteristics on four circular plots of 6 m in diameter, equally distributed along the stream and their midpoint being in a distance of 7 m to the stream edge. These variables were the number of *shrubs *(≤ 5 cm stem diameter at eye level) and the number of *trees *(> 5 cm), *canopy cover *(%; estimated), and *leaf litter *depth (cm; measured at 4 × 4 points and averaged). To measure riparian structural complexity, we took four photographs of the riparian vegetation of the stream, equally distributed along the 30 m of the stream section and alternating on the left and right side of the stream. The photographs covered an area of 3 × 2.25 m, with the bottom of each picture at the level of the water surface. *riparian vegetation *was then evaluated by projecting eight vertical and six horizontal grid lines onto the photograph, and measured by the number of tree structures such as branches and leaves at cross points of the grid lines.

### Species sampling, identification, and traits

We sampled tadpoles and invertebrates using dip nets of different sizes and materials, adjusted to obtain optimal sampling results for each stream. Sampling started downstream, and depending on stream width two to five people processed slowly on the same level upstream while dip-netting preferably all tadpoles and invertebrates in all microhabitats. We kept tadpoles alive and carried them in water containers in the laboratory. They were euthanized by immersion in chlorobutanol solution, and immediately sorted into series based on their morphology. From each series, we identified one specimen by DNA barcoding based on a fragment of the mitochondrial *16S *rRNA gene [39,40]. DNA sequences of all 1472 identified tadpole series (corresponding to 7020 individuals of 36 species, 2-18 species per stream) are deposited in Genbank (accession numbers FJ217329-FJ217345, GQ904717-GQ904746, GU808474-GU808492, GU974370-GU975745).

For all species present in the streams, we constructed a trait matrix based on morphological features that are known to be of ecological relevance (Table 1). By influencing basal resources and primary producers mainly due to foraging, tadpoles affect primary production and eventually stream ecosystem structure and function [31,32]. We therefore focused on ecological traits related to where and how tadpoles forage in the stream, and as a proxy for these we selected adequate morphological traits for analysis. We included features of oral disc shape, the shape of jaw sheaths, and presence of keratodonts as these traits are related to tadpole feeding [55,56]. We also used values for papillae and measurements of body shape as they are related with tadpole microhabitat [55,57,58].

### Statistical analyses

#### Ordination of environmental variables

We applied Principal Component Analysis (PCA) to reduce the dimensions of explanatory variables to a smaller set of orthogonal synthetic variables. We performed PCA on all 14 original habitat variables (see above) and for the 29 streams, on the correlation matrix in order to standardise for the influence of unequal variance. To evaluate data outliers and linear interdependence of variables, we used box-plots and pair-plots, respectively [59] (see additional file 2: data evaluation). As outliers can affect the outcome of the PCA, we reduced their influence by applying box-cox-power-transformations [60] on habitat variables containing outliers. An assumption of PCA is linearity and evaluating pair-plots, we found no obvious non-linear relation in the habitat variables. We assessed the significance of the PC loadings based on the bootstrapped-eigenvector method as suggested by Peres-Neto et al. [61]. We estimated the number of meaningful PCs by a scree plot [59]. We conducted multiple linear regressions with the first three PCs as independent variables and species richness as response variable (without interaction). Residuals of this and all other regression analyses were checked for patterns e.g. of heteroscedasticity, normality or highly influential data points using diagnostic plots (see additional file 3: diagnostic plots).

#### Species diversity

We assessed species richness (SR) in stream sections based on molecular identification of tadpoles sampled. We calculated functional diversity (FD) following the methodology of Petchey & Gaston [9,10]. This is a three-step dendrogram based classification function, in which a species trait matrix is used to calculate a pair-wise species distance matrix, which is used to construct dendrograms of specific species assemblages. The total branch length needed to connect all species in the assemblage represents the respective FD. There is a variety of distance measures and cluster methods available, however, there is no general rule of which methods perform best [62]. As our trait matrix, consisting of morphological traits of tadpoles (Table 1), contained both categorical and continuous variables, we used Gower's distance. We identified unweighted pair group method using arithmetic averages (UPGMA) as the best cluster method for our dataset, using an automated selection procedure implemented in the "GFD" script of Mouchet et al. [62]. GFD selects the combination of distance and clustering algorithms that best fits the species distribution in the functional trait space by minimizing the dissimilarity between the distance matrix and the ultrametric matrix of the functional tree. GFD calculates all the possible consensuses and simple dendrograms and selects the best by confronting them to the initial distance matrix. We applied polynomial regression of observed FD values as response and SR as independent variable to test for coherency and patterns of species redundancy (non linearity). For polynomial regression, higher powers of the explanatory variable are fitted to a linear model, and the significance of the new explanatory term is assessed by multiple regressions. Significant results show non-linearity in the data. To judge the level of FD of tadpole communities, we calculated a predicted value of FD for each observed community according to its SR level and compared these with the observed FD along a SR gradient. Each predicted FD value is the calculated mean of the FD of 500 randomly assembled communities. These were random assemblies chosen from all 36 species sampled in the study area, controlled for number of species. Due to non-normal distribution and violation of independence of residuals in the linear model for the predicted FD of the random communities, we used non-linear least-squares estimates of parameters of non-linear regression models describing an ascending asymptotic hyperbola (Michaelis-Menten kinetics). Such an asymptotic increase of FD can be expected by increasing SR in communities [9,49,51,63] and describes a FD-SR relationship with functional redundancy. Similar to an ANCOVA for linear relations, we used FD as the response variable, SR as the predictor and a binomial predicted-observed-variable as covariate (factor), and performed a t-test on the coefficients. We compared curve progression of observed and predicted FD data to access environmental impacts on species similarity within communities.

We used morphological traits, especially characters of oral disk and body shape [57] as proxies for resource use, including food and habitat choice of the tadpoles (Table 1) because no direct information on their diet and behaviour exists. Respective morphological data were sourced from publications [11,45,64-67]. If lacking a published description, they were assessed from the sampled specimens using a Zeiss StereoDiscovery microscope with Zeiss AxioVision software.

We performed all analyses in R 2.9.0 [68]. The code for bootstrapped-eigenvector method is courtesy of J. Oksanen. The Xtree function and further codes required for calculations of FD are courtesy of O. Petchey. The GFD code to identify the best distance measure and cluster method for FD calculation as well as their application following Mouchet et al. [62] was provided by the authors. In addition, the following packages were used during the analyses: car [69], clue [70], cluster [71], gtools [72], lattice [73], and nlme [74].

## Authors' contributions

AS participated in the design of the field study, conducted field work, designed and conducted statistical analyses, evaluated DNA barcoding results, and drafted the manuscript. ER conducted fieldwork and morphological analyses and contributed in discussions on the manuscript. RDR conducted fieldwork and conducted most of morphological measurements. MV designed the morphological and the DNA barcoding part and significantly developed the draft. JG designed the field study, conducted fieldwork, was substantially involved in the design of the statistical analyses and in the discussion of the results, and significantly developed the draft. All authors read and approved the final manuscript.

## Supplementary Material

Additional file 1 **regressions of PCs vs SR**. Plots for visual evaluation of the multiple regressions of Principal Components and species richness (SR). (A) to (C) display the regressions of SR depending on PC1 to PC3, respectively. PC3 was removed from the model and PC1 and PC2 remained. A summary of the correlation of SR and PC1 and PC2 is given in Figure 1 by a grey shading of the symbols. Click here for file

Additional file 2 **data evaluation**. Data evaluation of habitat variables used for Principal Component Analysis. Tadpole habitat characteristics are in lower case, adult frog habitat parameters are in capital letters. (A) Box-plots of the original and the transformed (with the extension "box-cox") habitat variables. We used these plots to evaluate data regarding outliers and extreme values that might influence the results of the PCA. We tried to minimise the influence of outliers on PCA by applying box-cox transformations on the original variables. Box-plots of transformed habitat variables are displayed next to the respective original habitat variable. (B) Pair plots of the habitat variables. We used these pair plots to evaluate data regarding strong non-linear relations between the habitat variables and extreme values in the multivariable space after data transformation. Click here for file

Additional file 3 **diagnostic plots**. Diagnostic plots used for model evaluation. Generally, panels 1 and 3 show residuals versus fitted values, panels 2 QQ-plots for normality, and panels 4 show standardised residuals vs. leverage and Cook statistics. (A) and (B) show diagnostic plots for the regression of observed functional diversity (FD) and species richness (SR) for the simple linear model (panels A1-4) and the quadratic model (panels B1-4). There were weak patterns in panels A1 and A2 which are reduced in panels B1 and B2. (C) and (D) show diagnostic plots for the regression of predicted FD of random communities and SR for the simple linear model (panels C1-4) and the quadratic model (panels D1-4). Note the very strong patterns in all C panels. Also in the quadratic model (D panels), strong patterns remain: D2 still shows non-normality in the residuals. Whereas D1 and D3 seem to show homogeneity in the data, they still show a violation of independence and D4 identifies highly influential points. We therefore desisted from applying a linear model on these data. (G) shows the residual plot to evaluate the non-linear regression of FD and SR. There is no obvious violation of homogeneity or independence. Click here for file
