# Supplementary figures and images for: The world's richest tadpole communities show functional redundancy and low functional diversity: ecological data on Madagascar's stream-dwelling amphibian larvae
Source: BMC Ecol. 2010 May 12;10:12. doi: 10.1186/1472-6785-10-12 (PMC2877654; doi:10.1186/1472-6785-10-12)

(A)

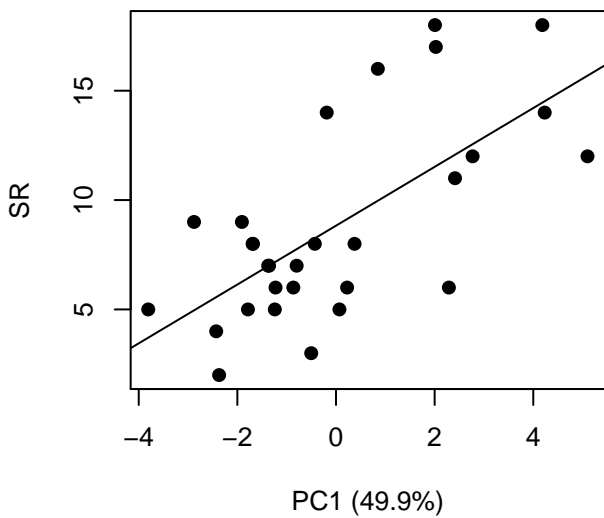

(B)

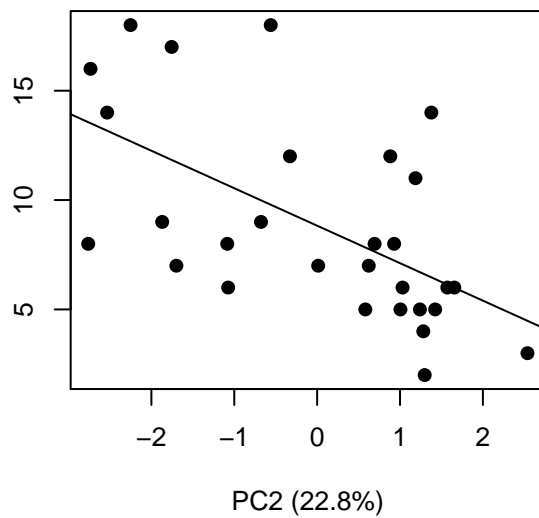

(C)

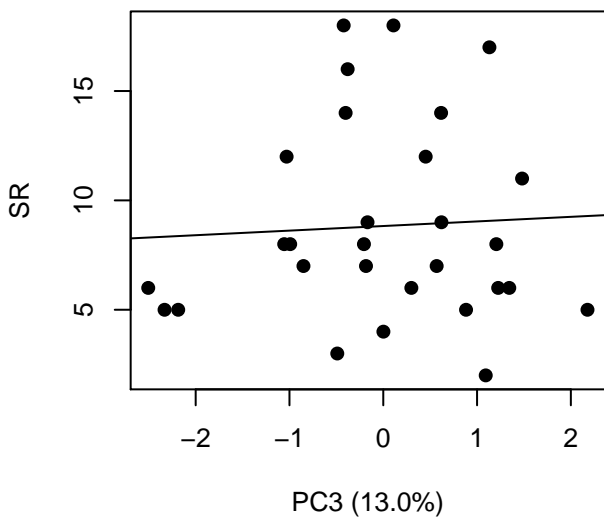

Supplement: Additional file 1 — regressions of PCs vs SR. Plots for visual evaluation of the multiple regressions of Principal Components and species richness (SR). (A) to (C) display the regressions of SR depending on PC1 to PC3, respectively. PC3 was removed from the model and PC1 and PC2 remained. A summary of the correlation of SR and PC1 and PC2 is given in Figure 1 by a grey shading of the symbols. [file 1472-6785-10-12-S1.PDF]
