# Supplementary material for: The world's richest tadpole communities show functional redundancy and low functional diversity: ecological data on Madagascar's stream-dwelling amphibian larvae
Source: BMC Ecol. 2010 May 12;10:12. doi: 10.1186/1472-6785-10-12 (PMC2877654; doi:10.1186/1472-6785-10-12)
Supplement: Additional file 2 — data evaluation. Data evaluation of habitat variables used for Principal Component Analysis. Tadpole habitat characteristics are in lower case, adult frog habitat parameters are in capital letters. (A) Box-plots of the original and the transformed (with the extension "box-cox") habitat variables. We used these plots to evaluate data regarding outliers and extreme values that might influence the results of the PCA. We tried to minimise the influence of outliers on PCA by applying box-cox transformations on the original variables. Box-plots of transformed habitat variables are displayed next to the respective original habitat variable. (B) Pair plots of the habitat variables. We used these pair plots to evaluate data regarding strong non-linear relations between the habitat variables and extreme values in the multivariable space after data transformation. [file 1472-6785-10-12-S2.PDF]

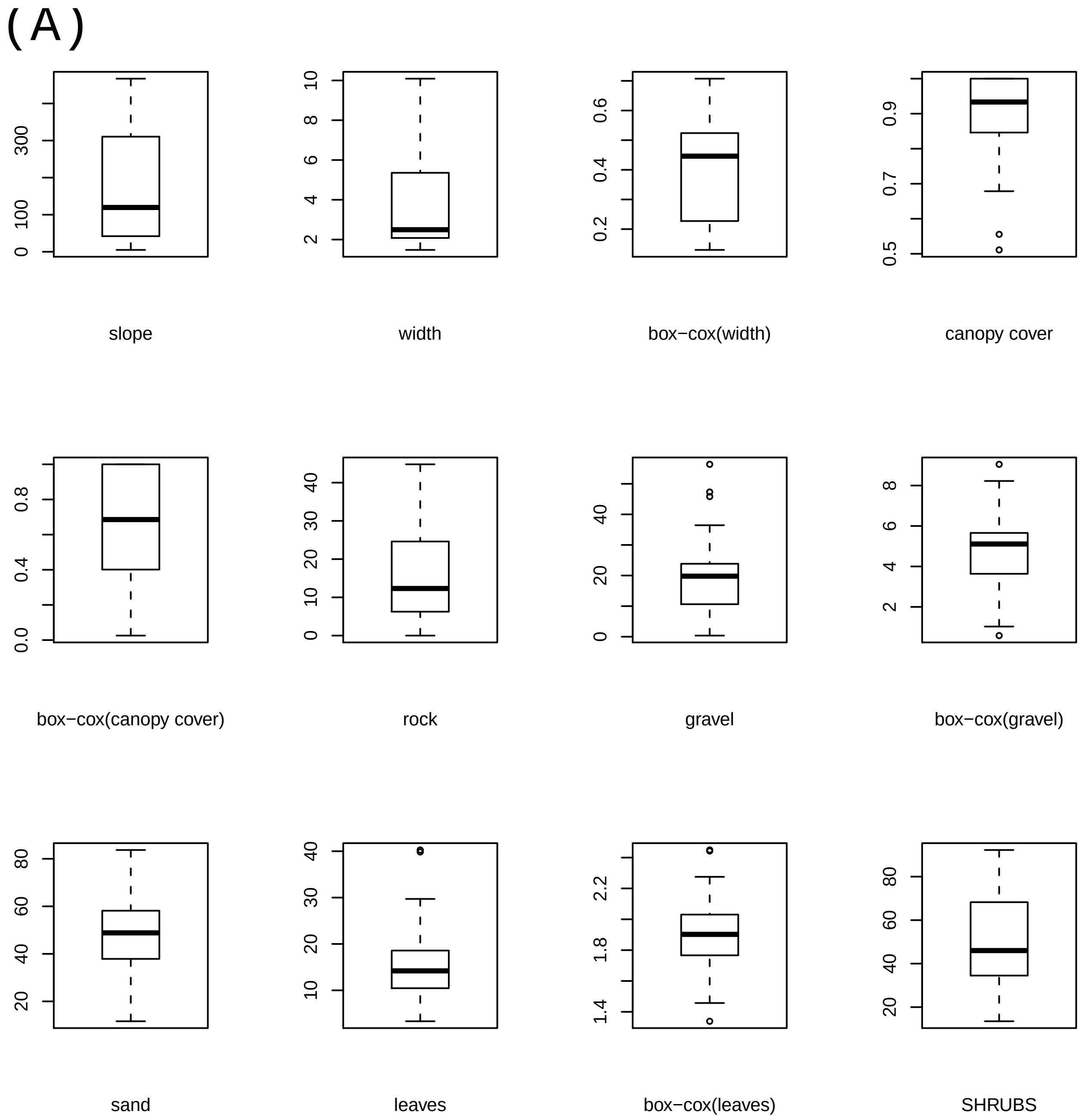
(A)
slope
width
box−cox(width)
canopy cover
box−cox(canopy cover)
rock
gravel
box−cox(gravel)
sand
leaves
box−cox(leaves)
SHRUBS

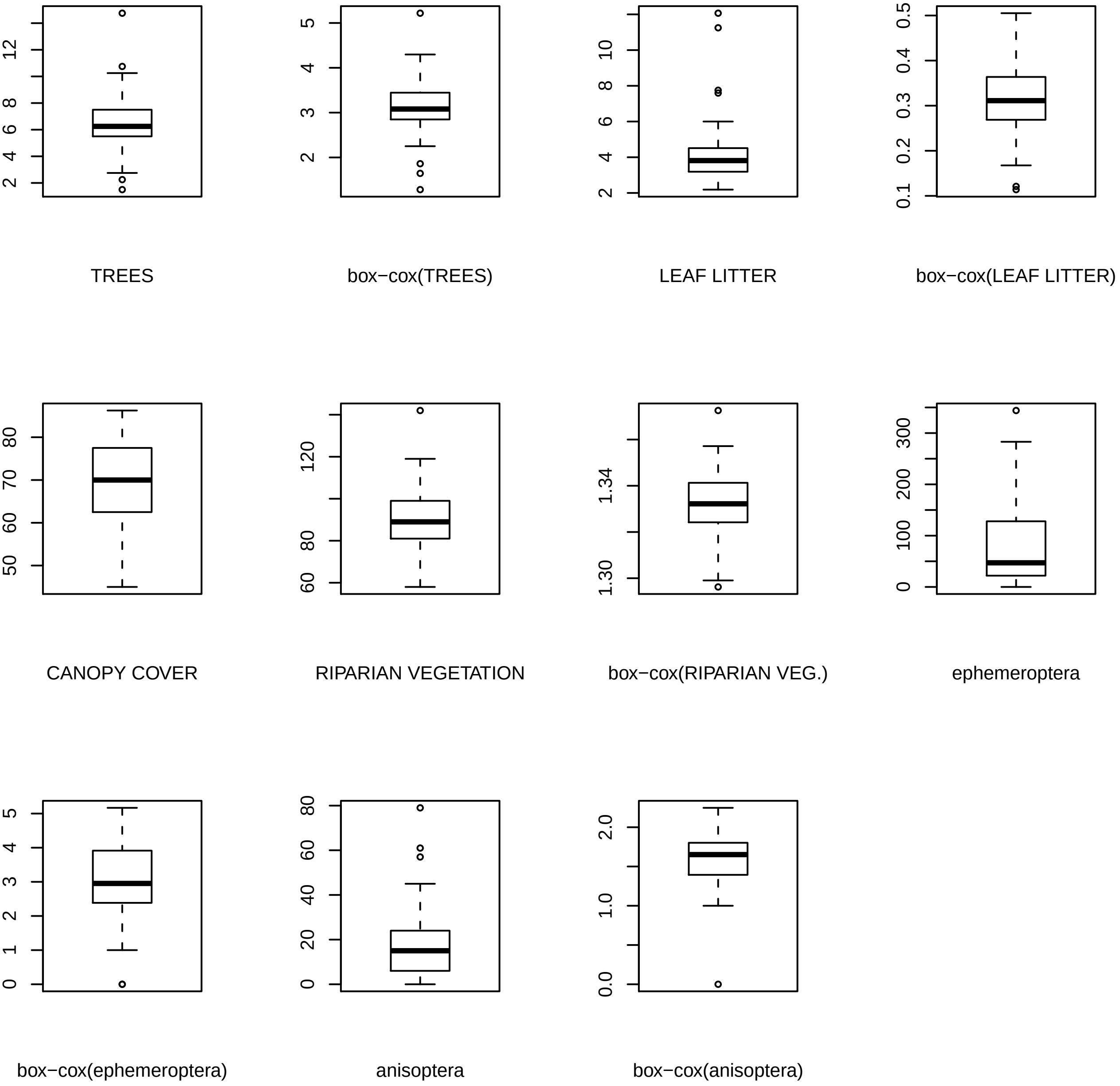
TREES
box−cox(TREES)
LEAF LITTER
box−cox(LEAF LITTER)
CANOPY COVER
RIPARIAN VEGETATION
box−cox(RIPARIAN VEG.)
ephemeroptera
box−cox(ephemeroptera)
anisoptera
box−cox(anisoptera)
2 4 6 8 12
2 3 4 5
2 4 6 8 10
0.1 0.2 0.3 0.4 0.5
50 60 70 80
60 80 120
1.30 1.34
0 100 200 300
0 1 2 3 4 5
0 20 40 60 80
0.0 1.0 2.0

(B)

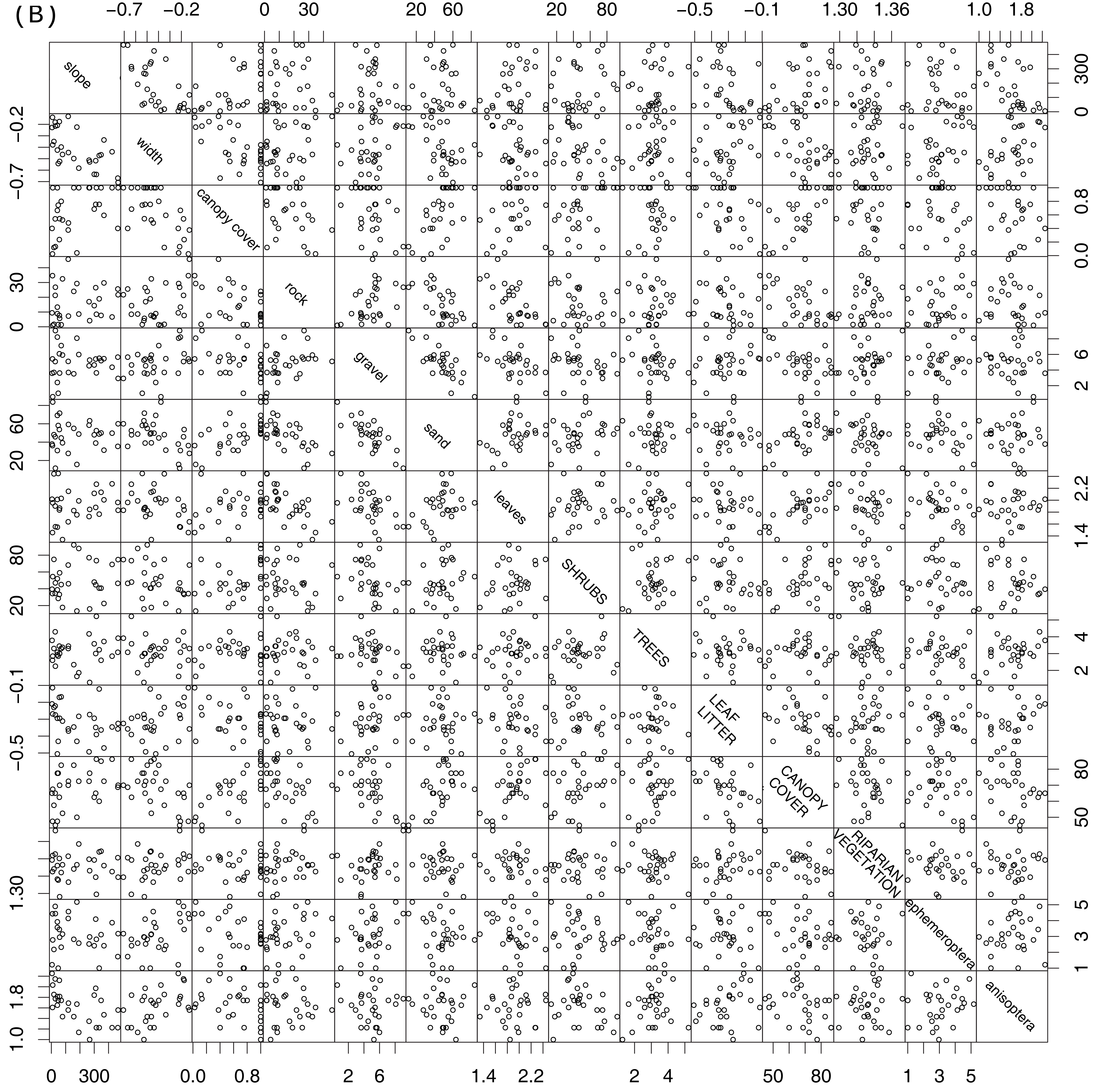
slope
width
canopy cover
rock
gravel
sand
leaves
SHRUBS
TREES
LEAF LITTER
CANOPY COVER
RIPARIAN VEGETATION
ephemeroptera
anisoptera
