# Supplementary material for: The world's richest tadpole communities show functional redundancy and low functional diversity: ecological data on Madagascar's stream-dwelling amphibian larvae
Source: BMC Ecol. 2010 May 12;10:12. doi: 10.1186/1472-6785-10-12 (PMC2877654; doi:10.1186/1472-6785-10-12)
Supplement: Additional file 3 — diagnostic plots. Diagnostic plots used for model evaluation. Generally, panels 1 and 3 show residuals versus fitted values, panels 2 QQ-plots for normality, and panels 4 show standardised residuals vs. leverage and Cook statistics. (A) and (B) show diagnostic plots for the regression of observed functional diversity (FD) and species richness (SR) for the simple linear model (panels A1-4) and the quadratic model (panels B1-4). There were weak patterns in panels A1 and A2 which are reduced in panels B1 and B2. (C) and (D) show diagnostic plots for the regression of predicted FD of random communities and SR for the simple linear model (panels C1-4) and the quadratic model (panels D1-4). Note the very strong patterns in all C panels. Also in the quadratic model (D panels), strong patterns remain: D2 still shows non-normality in the residuals. Whereas D1 and D3 seem to show homogeneity in the data, they still show a violation of independence and D4 identifies highly influential points. We therefore desisted from applying a linear model on these data. (G) shows the residual plot to evaluate the non-linear regression of FD and SR. There is no obvious violation of homogeneity or independence. [file 1472-6785-10-12-S3.PDF]

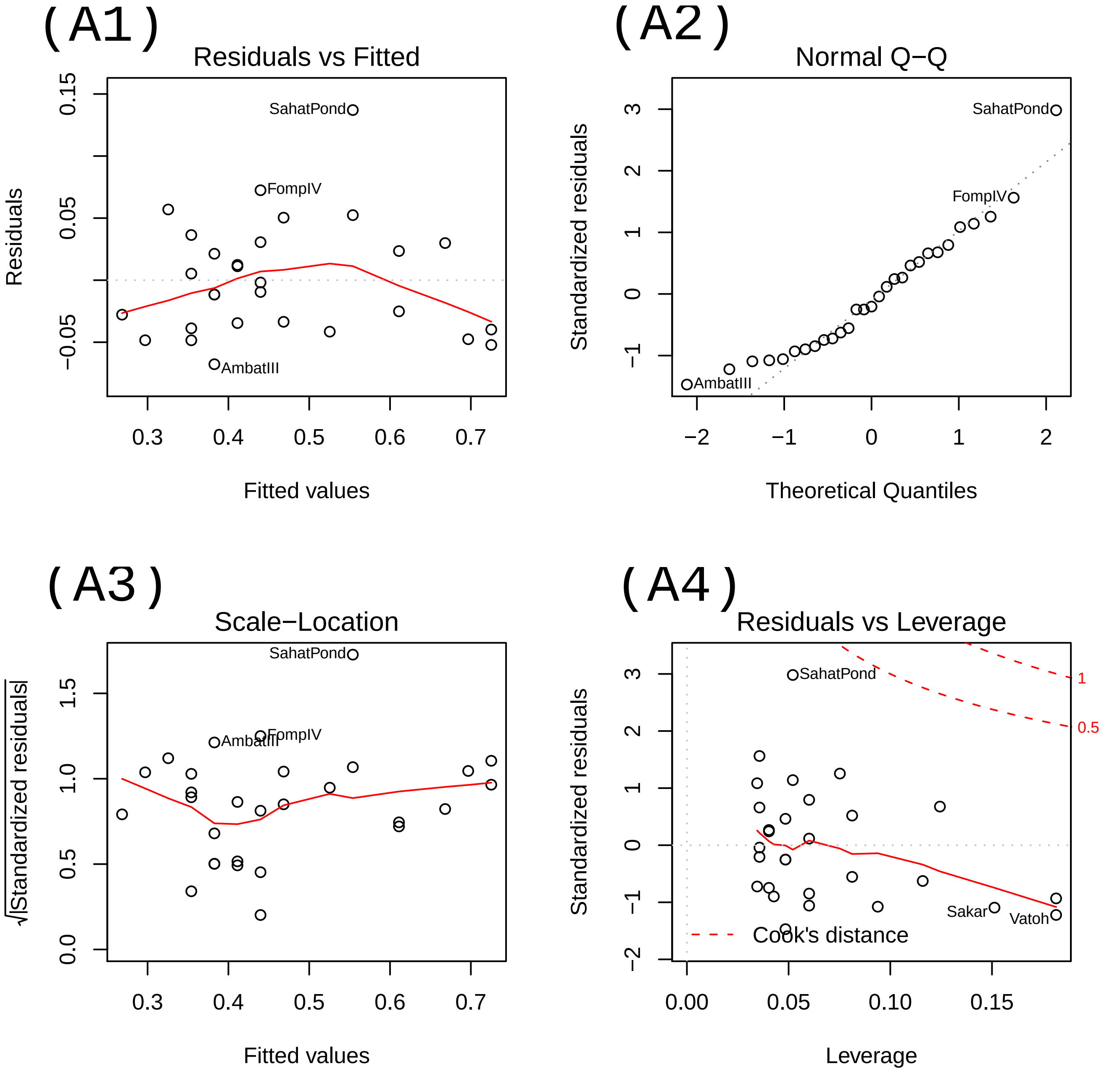
(A1)
Residuals vs Fitted
Residuals
Fitted values
0.15
0.05
−0.05
0.3
0.4
0.5
0.6
0.7
SahatPond
FompIV
AmbatIII
(A2)
Normal Q−Q
Standardized residuals
Theoretical Quantiles
3
2
1
0
−1
−2
SahatPond
FompIV
AmbatIII
(A3)
Scale−Location
√|Standardized residuals|
Fitted values
1.5
1.0
0.5
0.0
0.3
0.4
0.5
0.6
0.7
SahatPond
AmbatIII
FompIV
(A4)
Residuals vs Leverage
Standardized residuals
Leverage
3
2
1
0
−1
−2
0.00
0.05
0.10
0.15
SahatPond
Sakar
Vatoh
1
0.5
Cook's distance

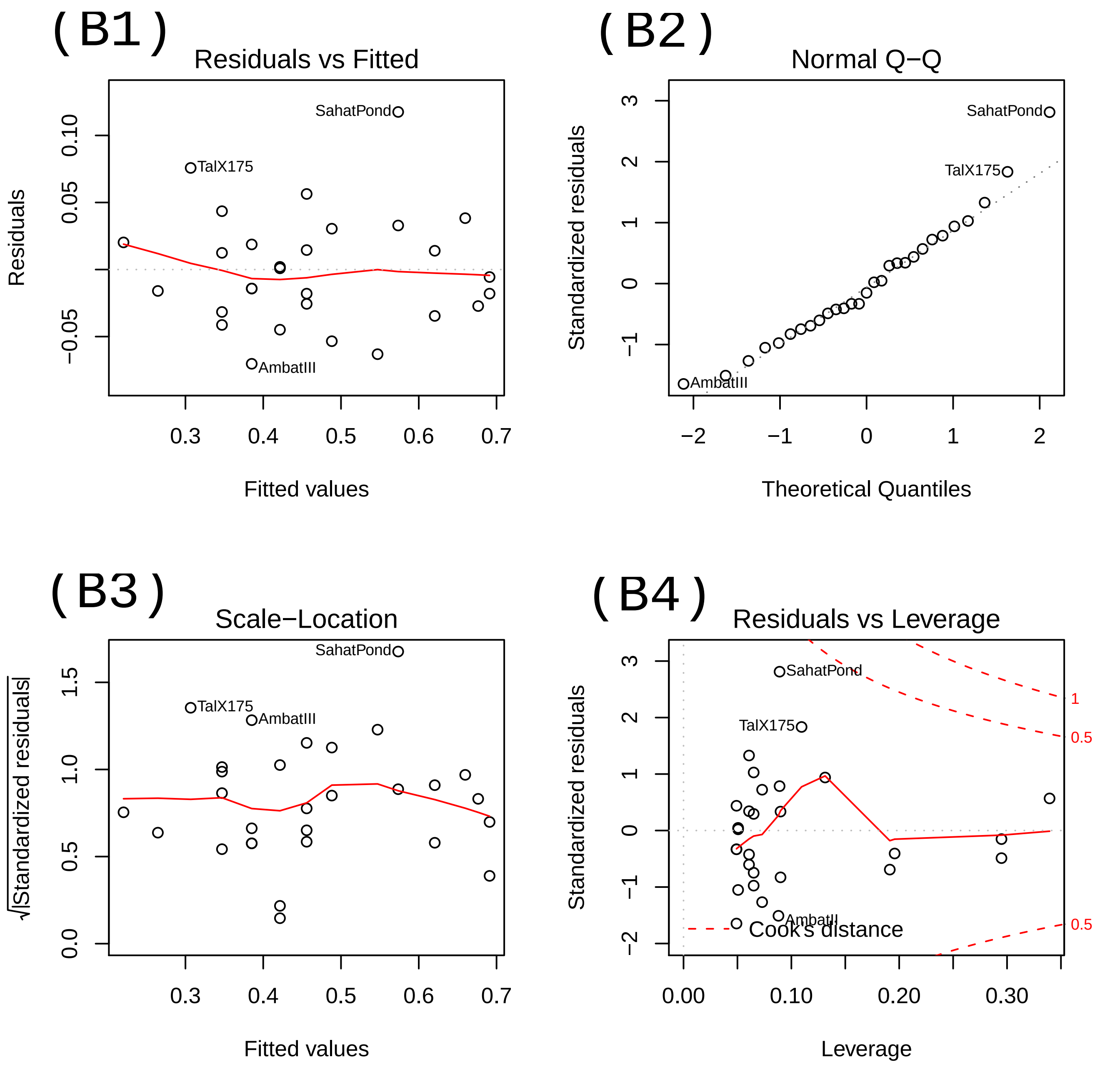

(B1)
Residuals vs Fitted
Residuals
Fitted values
SahatPond
TalX175
AmbatIII
(B2)
Normal Q−Q
Standardized residuals
Theoretical Quantiles
SahatPond
TalX175
AmbatIII
(B3)
Scale−Location
√|Standardized residuals|
Fitted values
SahatPond
TalX175
AmbatIII
(B4)
Residuals vs Leverage
Standardized residuals
Leverage
SahatPond
TalX175
AmbatIII
Cook's distance
1
0.5
0.5

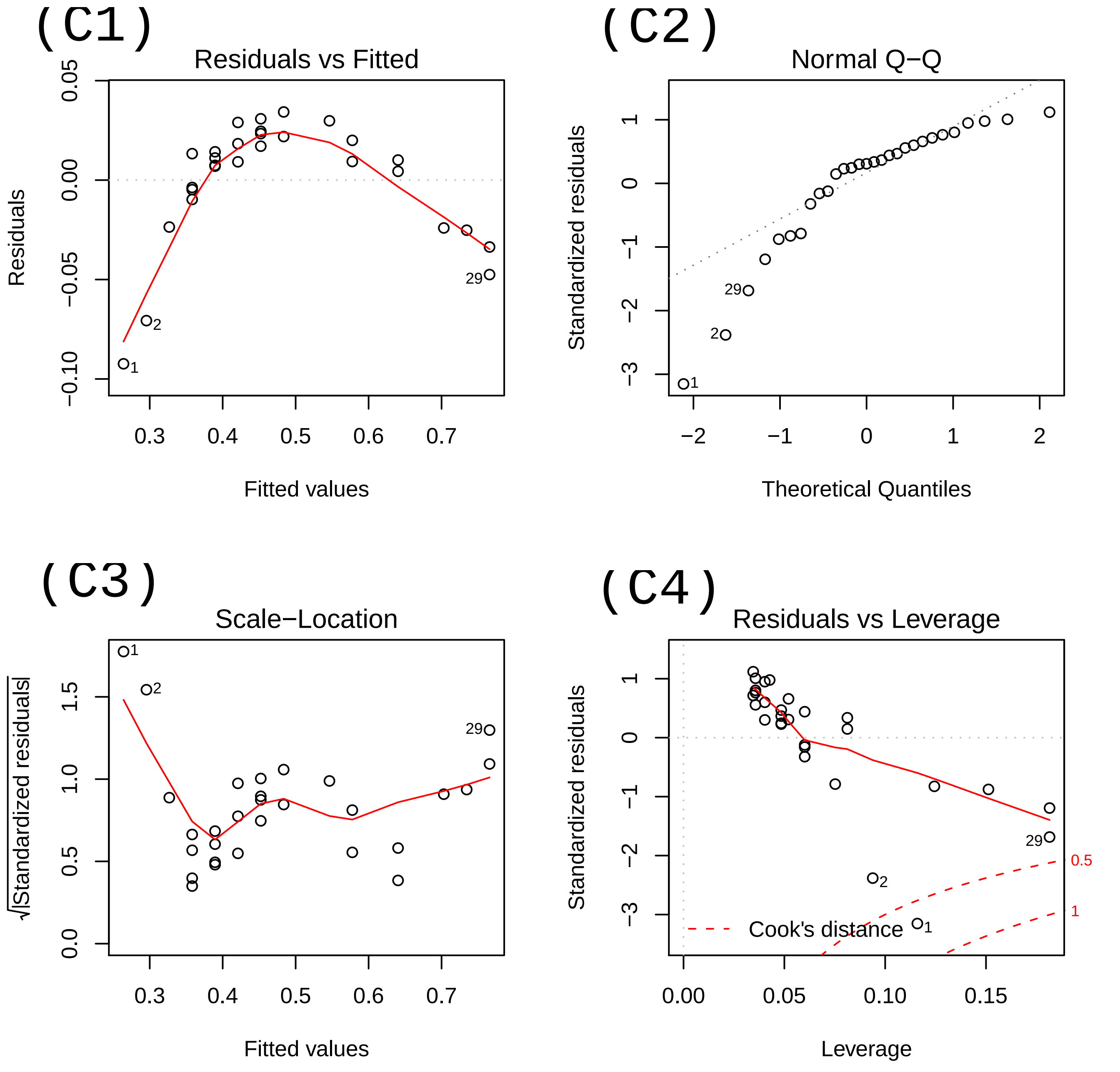
(C1)
Residuals vs Fitted
Residuals
Fitted values
0.05
0.00
−0.05
−0.10
0.3
0.4
0.5
0.6
0.7
1
2
29
(C2)
Normal Q−Q
Standardized residuals
Theoretical Quantiles
−2
−1
0
1
2
−3
1
2
29
(C3)
Scale−Location
√|Standardized residuals|
Fitted values
0.0
0.5
1.0
1.5
1
2
29
(C4)
Residuals vs Leverage
Standardized residuals
Leverage
0.00
0.05
0.10
0.15
Cook's distance
0.5
1
2
29

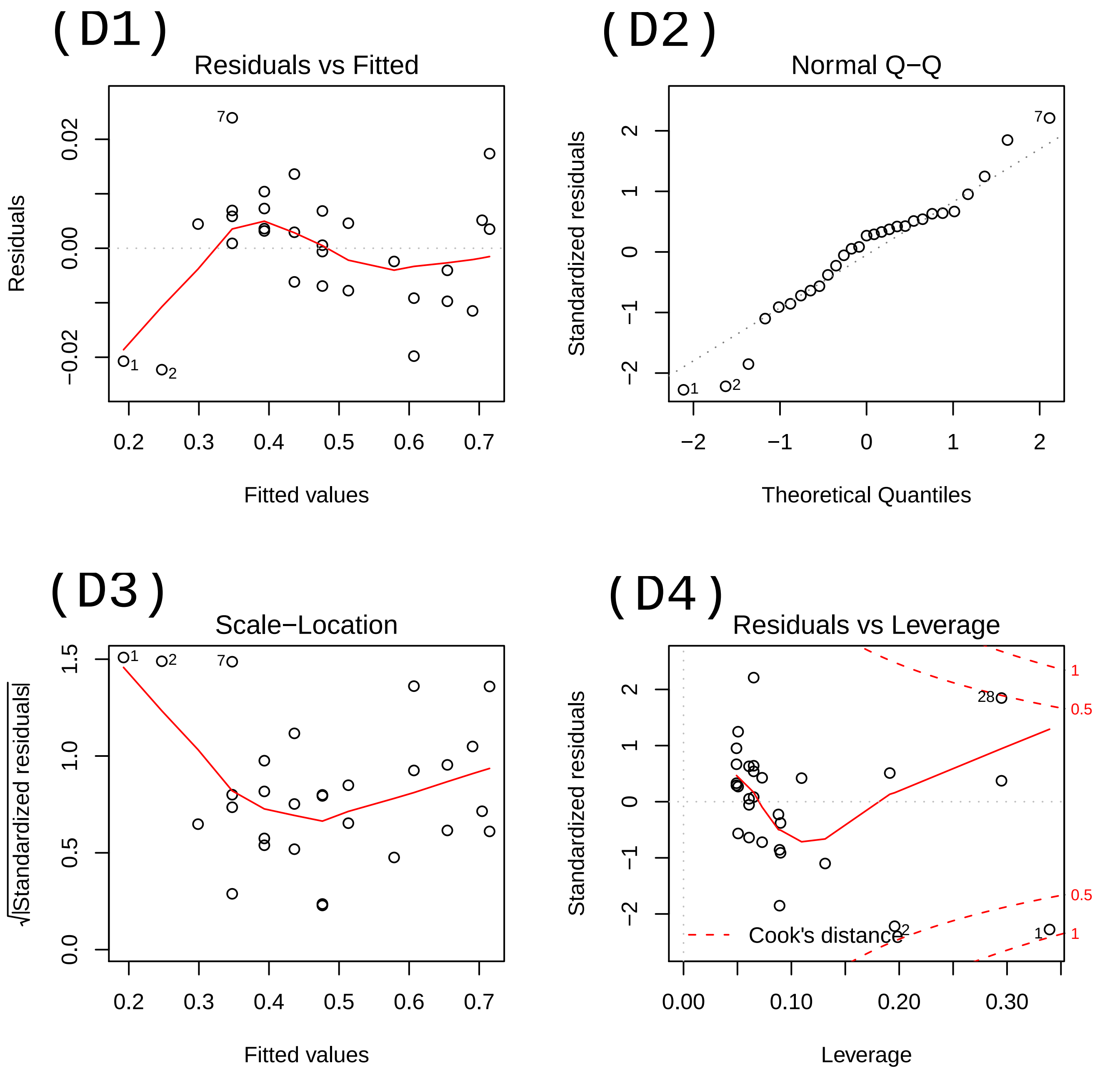
(D1)
Residuals vs Fitted
Residuals
Fitted values
0.02
0.00
−0.02
0.2
0.3
0.4
0.5
0.6
0.7
7
1
2
(D2)
Normal Q−Q
Standardized residuals
Theoretical Quantiles
−2
−1
0
1
2
7
1
2
(D3)
Scale−Location
√|Standardized residuals|
Fitted values
0.0
0.5
1.0
1.5
0.2
0.3
0.4
0.5
0.6
0.7
1
2
7
(D4)
Residuals vs Leverage
Standardized residuals
Leverage
−2
−1
0
1
2
0.00
0.10
0.20
0.30
Cook's distance
1
0.5
28
2
1

(E)

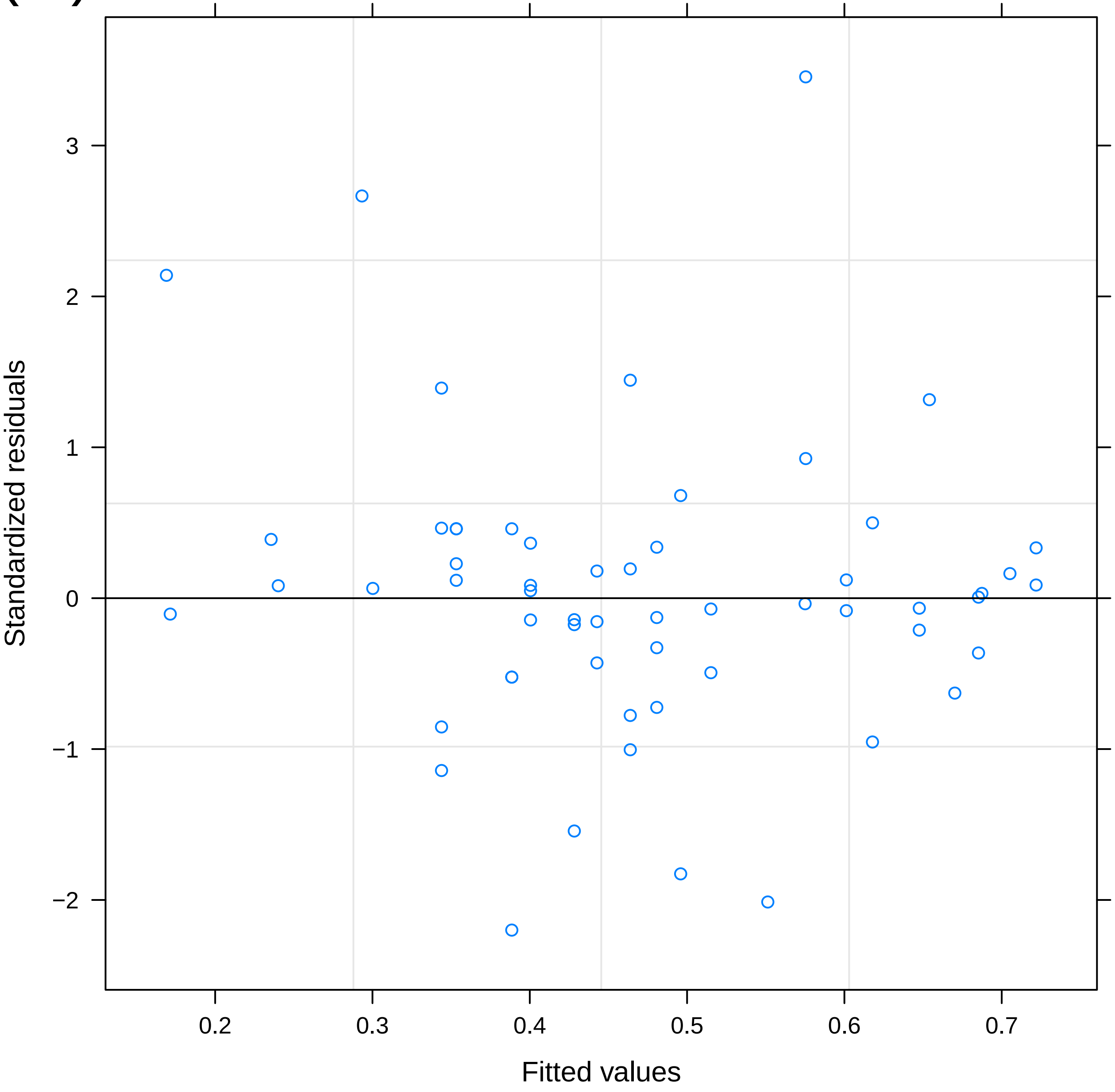

Standardized residuals
Fitted values
3
2
1
0
−1
−2
0.2
0.3
0.4
0.5
0.6
0.7
